# Knowledge, attitude, and practice towards enhanced recovery after surgery among colorectal cancer patients

**DOI:** 10.1038/s41598-024-59361-4

**Published:** 2024-04-19

**Authors:** Peng Xu, Da Li, Jian Li, Cheng Zhang

**Affiliations:** Department of General Surgery, General Hospital of Northern Theater Command (General Hospital of Shenyang Military Command), Shenyang, 110016 China

**Keywords:** Knowledge, Attitude, Practice, Colorectal cancer, Enhanced recovery after surgery, Cross-sectional study, Gastrointestinal cancer, Diseases

## Abstract

To explore the knowledge, attitude, and practice (KAP) towards enhanced recovery after surgery (ERAS) among colorectal cancer (CRC) patients. This cross-sectional study included CRC patients who underwent selective operation at the author’s Hospital, between April 2021 and April 2023. Their demographic characteristics and KAP towards ERAS were collected using a self-designed questionnaire. A total of 652 valid questionnaires were collected, with knowledge, attitude, and practice scores of 37.29 ± 11.35 (possible range: 11–55), 39.51 ± 6.40 (possible range: 11–55), and 6.53 ± 2.21 (possible range: 0–8), respectively. A positive correlation was found between knowledge and attitude (r = 0.291, P < 0.001), knowledge and practice (r = 0.292, P < 0.001), and attitude and practice (r = 0.363, P < 0.001). Structural equation model (SEM) analysis showed that knowledge had a significant direct effect on attitude (β = 0.164, P < 0.001) and attitude had direct effect on practice (β = 0.099, P < 0.001), indicating an indirect effect of knowledge on practice. Attitude also had a direct effect on practice (β = 0.038, P < 0.001). CRC patients showed moderate knowledge and attitude, and proactive practice towards ERAS. Further improvement of knowledge may improve their attitude and practice, leading to better outcomes and quality of care among CRC patients.

## Introduction

Colorectal cancer (CRC) is the third most common malignancy worldwide, causing significant morbidity and mortality^1^. It has been estimated that CRC-related incidence and deaths will substantially increase by 2030 due to aging populations, lifestyle factors such as smoking, alcohol consumption, obesity, lack of exercise and physical activities, dietary habits, family history, etc.^2,3^. At present, incidence rates are highest in developed countries but are increasing in developing countries. There are many treatment options for CRC patients^4^, and surgical intervention is the primary treatment option, however, it may significantly impact patients’ post-surgery outcomes^5^. Accordingly, perioperative nursing has a vital role in managing CRC patients throughout the perioperative period^6^, as it significantly impacts the prognosis of patients by ensuring a safe surgery, minimizes complications, provides required postoperative care, and promotes their education and advocacy^7^. Enhanced recovery after surgery (ERAS) protocols have been developed to address the impact of post-surgery outcomes in patients^8–10^. ERAS is a multi-dimensional approach and a combination of various peri-operative care methods aiming to reduce hospitalization time and surgical stress, maintain post-surgery physiological function, and enhance recovery rate after surgery^11–13^. This approach involves a multidisciplinary team approach that includes surgical, anesthetic, and nursing management, as well as patient education and participation.

A good understanding of ERAS could potentially enhance the cooperation and trust between patients and medical personnel. Attitude, which refers to patients' readiness, willingness, or reluctance to participate, is another key component for the successful implementation of ERAS among CRC patients^14^. Similarly, successful implementation of ERAS requires a structured and well-considered approach from all healthcare providers. The KAP analysis is a widely used research tool that has gained recognition within the medical field for its ability to determine the factors impacting behavioral change, such as education, work experience, level of medical facilities, ethical education, culture, and religious beliefs^15^. KAP analysis towards ERAS is crucial to understand CRC patients' position toward undergoing surgery and perioperative nursing. Good knowledge of ERAS enables the medical staff to identify the benefits, risks, and the need for preparation before the surgery. Attitude improves the perception and approach of the participants to issues like informed consent, autonomy, confidentiality, and dignity, while practice ensures the delivery of high-quality care^16^. KAP analysis among CRC patients can elucidate the gaps in their perception, thereby influencing their decision-making process about surgery^17^. This understanding can ultimately enhance the quality of ERAS and the creation of protocols for improving the outcomes.

While ERAS offers several benefits for improving the outcomes after surgery, the KAP of ERAS among CRC patients needed to be further investigated as it could have significant implications for improving the perception and attitude of CRC patients towards ERAS which may lead to better outcomes and quality of care. Therefore, this study aimed to explore the KAP of ERAS among CRC patients who underwent selective operation.

## Methods

### Study design and participants

The study was approved by the Ethics Committee of the author’s Hospital (Ethical approval ID: Y(2022)063), and all the participants signed an informed consent form. This cross-sectional study included CRC patients who underwent selective surgery at the author’s Hospital of Northern Theater Command in Shenyang, Liaoning, China, between April 2021 and April 2023. The inclusion criteria were: (1) pathology-confirmed diagnosis of CRC; (2) > 20 years old; and (3) the completion of radical resection (including procedures such as right and/or left hemicolon, total colon, subtotal colon, anterior rectum) with laparoscopic assistance. The exclusion criteria were: (1) the presence of serious mental illness; (2) severe liver failure, renal failure, or cardiovascular disease; (3) severe infectious or contagious diseases; and (4) questionnaires with short response times, incomplete responses, or inconsistent responses. After surgery, participants who met the inclusion criteria were sent the questionnaire electronically through *“Sojump”* platform (https://www.wjx.cn/vm/rXBoT59.aspx#) and the responses were collected following the checklist for reporting results of internet E-surveys (CHERRIES) guidelines^18^. The sample size for the study was calculated by using Cochran’s sample size estimation equation.

$$n= \frac{{z}^{2}pq}{{e}^{2}}$$

where ‘n’ represents the number of participants needed, with ‘z’ set to 1.96 for a 95% confidence interval, p = expected proportion, q = 1 − p, and ‘e’ representing a 5% margin of error. We chose 50% as the expected proportion to ensure capturing maximum sample size. By plugging these values into Cochran’s equation, we calculated that a total sample size of 384 participants are required for this study.

### Questionnaire

The questionnaire was designed by referring to the guidelines (*Chinese Expert Consensus on Accelerated Rehabilitation Surgery and Guidelines on Pathway Management*). During the pilot study with 51 patients, the face validity of the questionnaire was evaluated by determining if any items were unclear or confusing. Items that remained confusing even after explanation were subsequently removed. For construct validity, an analysis of Kaiser–Meyer–Olkin (KMO) and confirmatory factor analysis (CFA) of the questionnaire was conducted that was well aligned with the KAP model and demonstrated good construct validity (KMO = 0.952, P < 0.001; model fit indicators: CMIN/DF = 2.601, RMSEA = 0.050, IFI = 0.949, TLI = 0.945, CFI = 0.949) (Supplementary Table 1). Furthermore, a Cronbach's α = 0.827 score indicates a good internal consistency.

The final questionnaire was in Chinese that was translated to English language by professional translators and double checked for any errors or discrepancies at the time of interpretation of the results. The questionnaire included four dimensions: demographic characteristics (age, gender, body mass index [BMI], residence, ethnicity, education, the medical profession, monthly income, medical insurance, smoking, drinking, and underlying medical conditions), knowledge, attitude, and practice. The knowledge dimension consisted of 11 questions evaluated on a five-point Likert scale, ranging from 5 (very well comprehension) to 1 (very poorly comprehension). These questions mainly focus on different aspects of ERAS program, including the definition and main contents of ERAS, the necessity of post-operative recovery, assessment of nutrition, preoperative diet and bowel preparation requirements, the purpose of deep breathing and coughing, managing incisional pain, post-operative catheter retention, respiratory exercises, dietary requirements, and activity requirements. The attitude dimension also contained 11 questions that were evaluated on a five-point Likert scale, ranging from 5 (strongly agree) to 1 (strongly disagree). These questions mainly focus on various aspects of peri-operative care and recovery, including the benefits of ERAS protocols, education provided by medical staff, fasting and laxative use before surgery, nutritional support, pain management, catheter removal, respiratory training, water intake, and early mobilization to promote post-operative recovery. The practice dimension contained 8 questions, where 1 point was assigned for a ‘Yes’, and a 0 for a ‘No’ or ‘No impression’ response. These questions ask about the willingness of patients to cooperate with medical staff and follow guidelines related to ERAS, receiving necessary nutritional support, respiratory training, fasting suggestions, understanding pain management, resuming water intake and activities as advised post-operation, and receiving thromboprophylaxis as recommended. Participants’ knowledge, attitude, and practice were categorized based on the percentage of the total score. Scoring below 60% of total score indicated inadequate knowledge, negative attitude, or insufficient practice. Scoring between 60 and 80% of total score represented moderate knowledge, attitude, or practice. Scoring above 80% of total score indicated adequate knowledge, positive attitude, or proactive practice.

### Statistical analysis

Statistical analysis was conducted using SPSS 26.0 (IBM Corporation, Armonk, NY, USA) and Stata 17.0 (College Station, TX). Continuous variables that confirmed to normal distribution were expressed as mean ± standard deviation (SD), and compared by one-way analysis of variance (ANOVA) or independent-samples T test. And categorical variables were expressed as n (%). Correlations between participants' K, A, and P scores were analyzed using Pearson correlation analysis and the structural equation model (SEM). The SEM was based on the following assumptions: H1: The medical profession and education had positive effect on knowledge. H2: The knowledge had positive effect on attitude. H3: The attitude had positive effect on practice. H4: The knowledge had positive effect on practice. All tests were two-sided, with P < 0.05 indicating statistical significance.

### Ethics approval and consent to participate

All procedures were performed in accordance with the ethical standards laid down in the 1964 Declaration of Helsinki and its later amendments. The study was approved by the Ethics Committee of the General Hospital of Northern War Zone (Y (2022)063), and all the participants signed an informed consent form. All methods were carried out in accordance with relevant guidelines and regulations.

## Results

The questionnaire was distributed among 702 CRC patients, after excluding those refused to participate (n = 5, 4.64%), with conflict responses (n = 2, 1.85%), with repeated responses (n = 23, 21.36%), and response not within the time limit (n = 20, 18.57%), 652 valid questionnaires were collected. The participants included in our study met the requirement of calculated sample size. Of them, 230 (35.28%) aged 41–49 years old, 347 (53.22%) were male, 413 (63.34%) with urban residence, and 364 (55.83%) had education of college and above. The participant showed knowledge, attitude, and practice scores of 37.29 ± 11.35, 39.51 ± 6.40, and 6.53 ± 2.21, respectively (Table 1).

**Table 1:** Demographic characteristics and knowledge, attitude, and practice.

Variables	N (%)	Knowledge score		Attitude score		Practice score	
		Mean ± SD	P	Mean ± SD	P	Mean ± SD	P
Total	652	37.29 ± 11.35		39.51 ± 6.40		6.53 ± 2.21	
Age, years old			0.004		0.656		0.125
≤ 40	220 (33.74)	38.62 ± 10.90		40.35 ± 7.11		6.67 ± 2.08	
41–49	230 (35.28)	37.90 ± 11.67		38.81 ± 6.22		6.29 ± 2.39	
≥ 50	202 (30.98)	35.16 ± 11.23		39.39 ± 5.66		6.64 ± 2.13	
Gender			0.962		0.037		0.250
Male	347 (53.22)	37.31 ± 11.23		39.61 ± 6.05		6.62 ± 2.11	
Female	305 (46.78)	37.27 ± 11.51		39.39 ± 6.77		6.42 ± 2.32	
BMI, kg/m^2^			< 0.001		0.005		0.026
< 18.5	239 (36.66)	39.83 ± 10.67		38.92 ± 6.33		6.29 ± 2.27	
18.5–24.9	318 (48.77)	36.13 ± 11.54		39.39 ± 3.49		6.56 ± 2.17	
≥ 25	95 (14.57)	34.80 ± 11.32		41.39 ± 5.96		7.01 ± 2.14	
Residence			0.100		0.012		0.177
Urban	413(63.34)	36.74 ± 11.19		39.99 ± 6.52		6.62 ± 2.16	
Non-Urban	239(36.66)	38.26 ± 11.59		38.68 ± 6.09		6.37 ± 2.30	
Ethnicity			0.375		0.347		0.118
Han	599(91.87)	37.18 ± 11.34		39.44 ± 6.47		6.49 ± 2.23	
Ethnic minority	53(8.13)	38.62 ± 11.58		40.30 ± 5.45		6.98 ± 1.96	
Education			0.463		0.440		0.557
High school and below	288 (44.17)	36.93 ± 11.39		39.29 ± 5.88		6.58 ± 2.07	
Junior college and above	364 (55.83)	37.59 ± 11.33		39.68 ± 6.77		6.48 ± 2.32	
Medical profession			< 0.001		< 0.001		< 0.001
Yes	70 (10.74)	41.83 ± 11.37		43.21 ± 6.70		7.64 ± 1.50	
No	582 (89.26)	36.75 ± 11.24		39.06 ± 6.22		6.39 ± 2.25	
Monthly income (US dollars)			0.690		0.406		0.007
< 700	376 (57.67)	37.35 ± 11.61		39.22 ± 6.03		6.31 ± 2.31	
700–1400	203 (31.13)	37.56 ± 10.84		39.90 ± 6.83		6.72 ± 2.07	
> 1400	73 (11.20)	36.25 ± 11.49		39.92 ± 6.99		7.08 ± 1.96	
Medical insurance			0.282		0.857		0.999
Insured	557 (85.43)	37.10 ± 11.29		39.53 ± 6.64		6.53 ± 2.25	
Uninsured	95 (14.57)	38.45 ± 11.73		39.40 ± 4.74		6.53 ± 1.97	
Smoking			0.750		0.686		0.298
Yes	210 (32.21)	37.50 ± 11.71		39.36 ± 6.38		6.40 ± 2.21	
No	442 (67.79)	37.20 ± 11.19		39.58 ± 6.41		6.59 ± 2.21	
Drinking			0.155		0.054		0.012
Yes	316 (48.47)	37.95 ± 11.43		39.01 ± 6.10		6.30 ± 2.30	
No	336 (51.53)	36.68 ± 11.26		39.98 ± 6.64		6.74 ± 2.11	
Underlying medical conditions			0.085		0.293		0.784
Yes	316 (48.47)	38.09 ± 11.27		39.24 ± 6.54		6.55 ± 2.18	
No	336 (51.53)	36.55 ± 11.40		39.76 ± 6.25		6.50 ± 2.24	

*SD* standard deviation, *BMI* body mass index.

The knowledge scores were likely to be higher in participants aged ≤ 40 years old, with lower BMI, being medical profession (all P < 0.05) (Table 1). The highest percentage of participants who reported comprehending a question very well was for the purpose of deep breathing and coughing up sputum before surgery (23.93%). In contrast, 34.51% of participants had a relatively high level of comprehension regarding the preoperative diet requirements for ERAS (34.51%) (Supplementary Table 2).

The attitude was likely to be more positive in participants with male gender, with higher BMI, with urban residence, and being medical profession (all P < 0.05) (Table 1). The response showed that the majority of participants agreed or strongly agreed that undergoing ERAS allowed them to recover soon after surgery (Q1, 68.56%), and they found information and education provided by the medical staff in the peri-operative period helpful (Q2, 71.78%). Most participants disagreed or strongly disagreed that not taking oral laxatives (Q3, 59.12%) and fasting (Q4, 56.44%) before surgery would not affect their surgery. The majority of participants agreed or strongly agreed that deep breathing and coughing up sputum (Q5, 63.19%) and receiving nutritional support (Q6, 75.07%) before surgery would be helpful for them. The majority of participants agreed or strongly agreed that awareness of pain scores and correct usage of analgesic pumps and medication (Q7, 66.10%) and removal of catheter after surgery (Q8, 64.73%) would be useful for relieving incisional pain and wound recovery, respectively. Most participants agreed or strongly agreed that postoperative respiratory training (Q9, 68.71%) and resumption of water intake (Q10, 60.89%) would help cough up sputum and increase oral comfort, respectively. Most participants (Q11, 73.47%) agreed or strongly agreed that getting out of bed and moving around in the early postoperative period is beneficial to the recovery of bowel function and thrombosis prevention (Supplementary Table 3).

The practice scores were likely to be higher in participants with higher BMI, being medical profession, had higher monthly income, and had no history of drinking (all P < 0.05) (Table 1). The percentage of participants who answered ‘Yes’ to each practice ranged from 77.61 to 85.28% (Supplementary tables 4).

**Figure 1:**
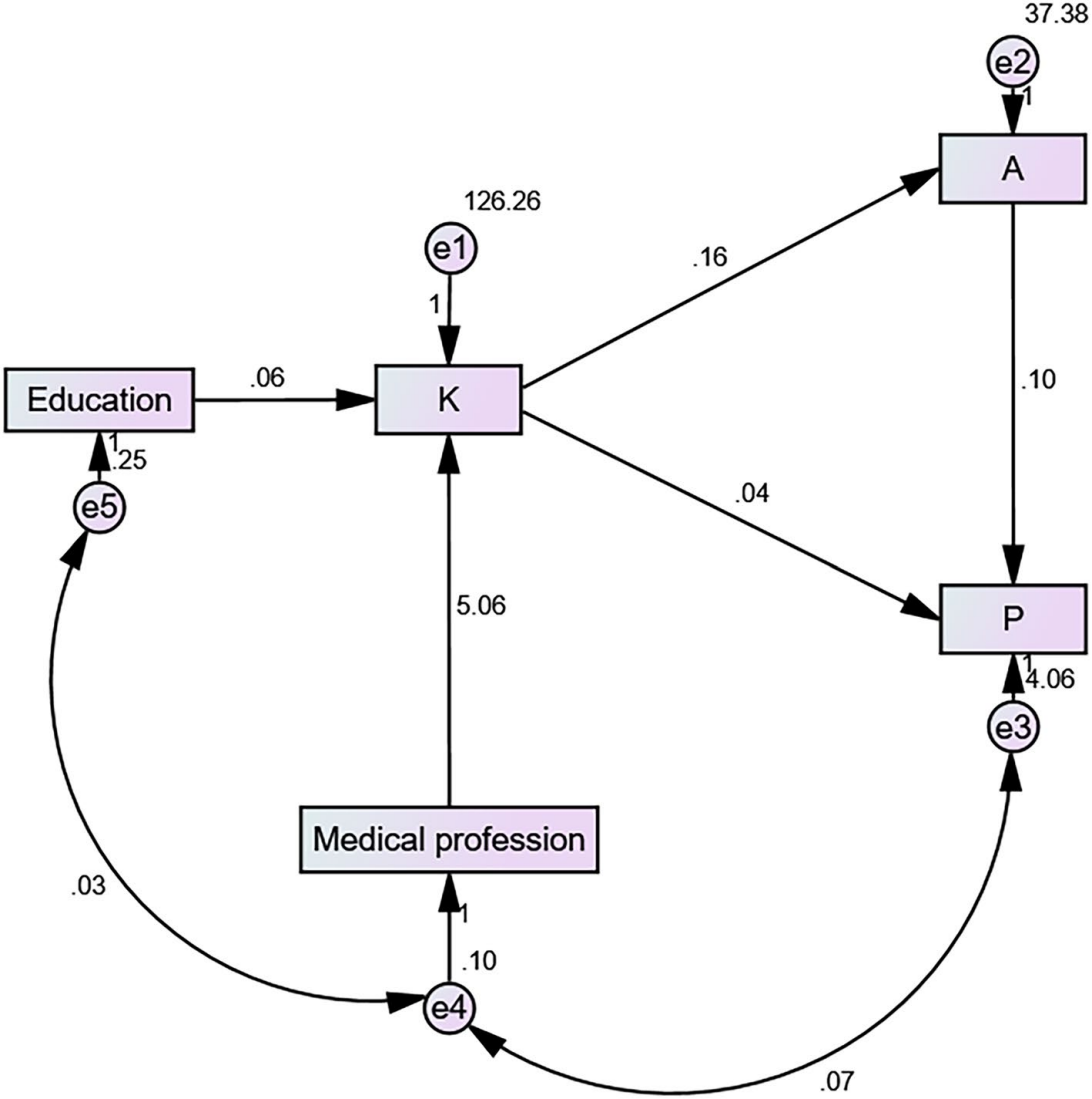
Illustration of structural equation model (SEM) analysis.

A positive correlation was found between knowledge and attitude (r = 0.291, P < 0.001), knowledge and practice (r = 0.292, P < 0.001), and attitude and practice (r = 0.363, P < 0.001) (Table 2). The SEM analysis showed that the medical profession had a direct effect on knowledge (estimate = 5.060, P < 0.001), knowledge had a significant direct effect on attitude (estimate = 0.164, P < 0.001) and attitude had direct effect on practice (estimate = 0.099, P < 0.001), indicated an indirect effect of knowledge on practice. Attitude also had a direct effect on practice (estimate = 0.038, P < 0.001) (Table 3 and Fig. 1).

**Table 2:** Results of correlation analysis.

	Knowledge	Attitude	Practice
Knowledge	1		
Attitude	0.291 (P < 0.001)	1	
Practice	0.292 (P < 0.001)	0.363 (P < 0.001)	1

**Table 3:** Results of structural equation model (SEM) analysis.

			Estimate	P
K	< –	Medical profession	5.060	< 0.001
K	< –	Education	0.063	0.945
A	< –	K	0.164	< 0.001
P	< –	K	0.038	< 0.001
P	< –	A	0.099	< 0.001

## Discussion

This study showed CRC patients underwent selective surgery had moderate knowledge and attitude and a proactive practice towards ERAS, the result also revealed the positive correlation among their KAP. The findings might have implications for improving the perception and attitude of CRC patients towards ERAS, eventually leading to better outcomes and quality of care.

A clear understanding of ERAS by the patients enables them to identify the benefits, risks, and the need for preparation before surgery. The findings of this study regarding the impact of demographic characteristics on ERAS among CRC patients are consistent with previous research. For instance, age was significantly associated with knowledge scores about ERAS, with younger patients (aged ≤ 40) having the highest mean knowledge score. This could be because younger patients can access health information through various sources, such as the Internet and social media. This finding is consistent with previous research showing that younger individuals have higher health literacy levels and are more likely to actively seek health information^19,20^. Gender was also significantly associated with attitude score towards ERAS, with females having a slightly lower mean attitude score than males. Societal and cultural factors may shape gender differences in attitudes toward ERAS. Previous studies have also suggested that women may have different healthcare-seeking behaviors and attitudes toward medical treatments than men^21,22^. BMI was found to be significantly associated with all three scores of ERAS, suggesting that individuals with different BMI levels have different levels of KAP toward ERAS. A previous study has shown that individuals with higher BMI may have a higher risk of complications and slower recovery after surgery^23^. It is possible that individuals with higher BMI are more motivated to learn about and adopt practices that promote faster recovery and better outcomes. Urban residents had higher scores in attitude and practice of ERAS compared to non-urban residents, likely due to differences in access to healthcare resources, leading to a greater understanding and adoption of ERAS practices. Several factors contribute to poorer education in rural areas such as limited resources, lack of access to educational opportunities, poverty, limited transportation, cultural factors, etc. Education was not significantly associated with any of the scores, which was somewhat surprising and inconsistent with previous research reporting a positive association between education and health knowledge^24^. It is possible that the sample population in this study had a relatively overall high level of education, thus minimizing the differences in knowledge, attitude, and practice scores between different education levels. The medical profession and medical insurance were both significantly associated with all three scores of ERAS, which suggests that individuals with a medical background or healthcare coverage may have better knowledge, better attitude, and practice regarding ERAS. This finding is in line with previous research that has shown a positive association between healthcare access and health literacy^19,20^. Monthly income was significantly associated with practice scores of ERAS, with higher-income individuals having higher mean practice scores. This finding is consistent with previous research showing a positive association between socioeconomic status and health behaviors^25^. Patients with higher incomes may have more resources and support to engage in practices promoting faster recovery after surgery.

The present study also assessed CRC patients' knowledge, attitude, and practice regarding ERAS. The results showed varying levels of knowledge about different aspects of the surgery among the participants. The highest level of knowledge was reported for the purpose of deep breathing and coughing up sputum before surgery, which may be because these practices are commonly recommended by healthcare providers before surgery^26,27^. However, the lowest level of knowledge was reported for the requirements of post-surgery catheter retention, which suggests that healthcare providers need to focus more on educating patients about the importance and care related to post-surgery catheter management. The study findings indicate that CRC patients generally had a positive attitude towards ERAS protocols, which can help accelerate their recovery. Most participants agreed with the benefits of surgery and various pre- and post-surgery practices. This positive attitude may stem from perceived benefits such as faster recovery and improved quality of life after surgery. Additionally, clear communication and education from healthcare providers about the benefits of ERAS may have played a role in shaping the attitude of patients towards these protocols. It is strongly recommended that healthcare providers continue to promote ERAS protocols to optimize outcomes and support patients in their recovery process. The practice of ERAS among CRC patients was generally high, with a high percentage of participants reporting adherence to the recommended practices as indicated by reduced postoperative gastric tube indwelling time (7.4 h), postoperative diet recovery time (50.2 h), anal exhaust time (70.2 h), postoperative time to get out of bed (20.5 h), and postoperative hospital stay (8.2 days)^28^. These findings are encouraging and suggest that patients are motivated to actively engage in practices that could promote their recovery. However, further research is needed to further validate these self-reported practices and assess their actual implementation.

The correlation analysis revealed moderate positive correlations between knowledge and attitude, knowledge and practice, and attitude and practice. These findings suggest that as ERAS-related knowledge increases, there is a likelihood of more positive attitudes and better practices among CRC patients. This highlights the importance of patient education and empowerment in improving outcomes and promoting adherence to recommended practices. These results are also consistent with a previous study^29^. SEM analysis further supported the relationship between KAP scores and predictor variables towards ERAS among CRC patients. Our results showed that the medical profession had a significant negative effect on the knowledge about ERAS after surgery, indicating a tendency toward overestimation of the relevant knowledge and reluctance to learn more about the disease^30,31^. Overall, SEM results suggested that knowledge about CRC is an important predictor of attitudes toward and preparation to care for people with the disease and that the medical profession may not necessarily imply greater knowledge about the disease. This also suggests the need for targeted educational interventions for individuals working in healthcare to enhance their knowledge about ERAS.

The present study has a few limitations. First, the study was limited to a single hospital, thus limiting the generalizability of the findings to other populations and settings. Second, the study only included CRC patients who chose the appropriate timing for surgery, which may introduce selection bias and limit the representativeness of the study sample. Third, the cross-sectional study design limited the ability to establish causal relationships between variables and only provided insight into the KAP scores of participants at a specific time point. Fourth, the study included limited demographic characteristics as potential confounding factors and did not explore other factors that may influence KAP scores, such as socioeconomic status, social support, or health literacy.

In conclusion, the study found that age, gender, BMI, residence, education level, and occupation significantly impacted the knowledge, attitude, and practice scores of CRC patients regarding ERAS protocols. Overall, participants demonstrated a positive attitude towards ERAS, with higher knowledge scores leading to better adherence to recommended practices. The medical profession was also found to play a crucial role in educating patients about ERAS protocols, ultimately impacting their attitude and practice. The study underscores the importance of patient education and healthcare professional involvement in promoting and implementing ERAS protocols for improved outcomes in CRC patients undergoing surgery The study further highlights the importance of improving knowledge and awareness about ERAS among CRC patients to achieve better outcomes and quality of care. Further research and interventions are warranted to address the gaps in this study and enhance the overall management of CRC patients undergoing ERAS.

### Supplementary Information

Supplementary Tables.

## Data Availability

All data generated or analysed during this study are included in this published article [and its supplementary information files].

## Abbreviations

KAP: Knowledge, attitude, and practice
CRC: Colorectal cancer
ERAS: Enhanced recovery after surgery
SEM: Structural equation model
BMI: Body mass index
CHERRIES: Checklist for reporting results of internet E-surveys
