# Supplementary material for: Knowledge, attitude, and practice towards enhanced recovery after surgery among colorectal cancer patients
Source: Sci Rep. 2024 Apr 19;14:9034. doi: 10.1038/s41598-024-59361-4 (PMC11031563; doi:10.1038/s41598-024-59361-4)
Supplement: Supplementary file 1 — Supplementary Tables. [file 41598_2024_59361_MOESM1_ESM.docx]

**Supplementary tables 1. Model fit indicators in confirmatory factor analysis**

| Indicators | Reference | Actual |
| --- | --- | --- |
| CMIN/DF | 1-3: Excellent, 3-5: Good | 2.601 |
| RMSEA | <0.08: Good | 0.050 |
| IFI | >0.8: Good | 0.949 |
| TLI | >0.8: Good | 0.945 |
| CFI | >0.8: Good | 0.949 |

**Supplementary tables 2.** Knowledge

| **Knowledge** | **N (%)** | | | | |
| --- | --- | --- | --- | --- | --- |
|  | **Very well comprehended** | **Relatively comprehended** | **Unsure** | **Not very well comprehended** | **Very poorly comprehended** |
| Q1. Are you familiar with the definition and main contents of ERAS? | 119(18.25) | 190(29.14) | 141(21.63) | 151(23.16) | 51(7.82) |
| Q2. Do you understand the importance of post-operative recovery in the context of ERAS? | 124(19.02) | 205(31.44) | 35(20.71) | 142(21.78) | 46(7.06) |
| Q3. How does your healthcare provider assess your nutrition in relation to ERAS? | 132(20.25) | 213(32.67) | 124(19.02) | 141(21.63) | 42(6.44) |
| Q4. What are the specific requirements for a preoperative diet in the ERAS protocol? | 132(20.25) | 225(34.51) | 124(19.02) | 134(20.55) | 37(5.67) |
| Q5. What are the preoperative bowel preparation requirements for patients undergoing ERAS? | 114(17.48) | 238(36.50) | 114(17.48) | 140(21.47) | 46(7.06) |
| Q6. What is the significance of deep breathing and coughing up sputum before undergoing ERAS? | 156(23.93) | 205(31.44) | 128(19.63) | 126(19.33) | 37(5.67) |
| Q7. What measures are taken to manage incisional pain in the ERAS protocol? | 112(17.18) | 205(31.44) | 143(21.93) | 145(22.24) | 47(7.21) |
| Q8. What are the requirements of post-operative catheter retention as part of ERAS? | 141(21.63) | 220(33.74) | 116(17.79) | 144(22.09) | 31(4.75) |
| Q9. What are the different methods of post-operative respiratory exercises recommended in the ERAS protocol? | 123(18.87) | 219(33.59) | 135(20.71) | 138(21.17) | 37(5.67) |
| Q10. What are the dietary requirements for post-operative exercises recommended in the ERAS protocol? | 125(19.17) | 221(33.90) | 129(19.79) | 137(21.01) | 40(6.13) |
| Q11. What are the activity requirements for patients during the post-operative phase of ERAS? | 148(22.70) | 225(34.51) | 120(18.40) | 126(19.33) | 33(5.06) |

**Supplementary tables 3.** Attitude

| **Attitude** | **N (%)** | | | | |
| --- | --- | --- | --- | --- | --- |
|  | **Strongly agree** | **Agree** | **Neutral** | **Disagree** | **Strongly disagree** |
| Q1. Did undergoing ERAS help you recover quickly after your operation? | 190(29.14) | 257(39.42) | 153(23.47) | 39(23.47) | 13(1.99) |
| Q2. Did the information and education provided by the medical staff during the peri-operative period assist you? | 199(30.52) | 269(41.26) | 127(19.48) | 41(6.29) | 16(2.45) |
| Q3. Do you believe that not taking oral laxatives before surgery has any impact on the outcome of the surgery? | 27(4.14) | 75(11.50) | 167(25.61) | 225(34.51) | 158(24.23) |
| Q4. Do you think that shortening the pre-operative fasting for water and food has any impact on the success of the surgery? | 33(5.06) | 82(12.58) | 169(25.92) | 214(32.82) | 154(23.62) |
| Q5. Will deep breathing and coughing before surgery aid in easier removal of sputum post-surgery? | 159(24.39) | 253(38.80) | 173(26.53) | 51(7.82) | 16(2.45) |
| Q6. Will receiving nutritional support before surgery improve your overall nutritional status? | 219(33.59) | 277(42.48) | 111(17.02) | 36(5.52) | 9(1.38) |
| Q7. Do you think understanding pain scores and utilizing analgesic pumps and medication properly will help alleviate incisional pain? | 175(26.84) | 256(39.26) | 156(23.93) | 50(7.67) | 15(2.30) |
| Q8. How do you feel about the healthcare provider's removal of the catheter or laparoscopic tube 1 to 2 days after surgery – does it cause my discomfort or complications in your opinion? | 186(28.53) | 236(36.20) | 164(25.15) | 54(8.28) | 12(18.84) |
| Q9. Do you believe that post-operative respiratory training can help with easier sputum removal? | 185(28.37) | 263(40.34) | 145(22.24) | 48(7.36) | 11(1.69) |
| Q10. Will resuming water intake shortly after surgery help with oral comfort, thirst relief, and promote bowel function recovery? | 151(23.16) | 146(37.73) | 180(27.61) | 63(9.66) | 12(1.84) |
| Q11. Do you agree that getting out of bed and moving around soon after surgery can aid in bowel function recovery and prevent thrombosis? | 212(32.52) | 267(40.95) | 126(19.33) | 38(5.83) | 9(1.38) |

[**Supplementary tables**](javascript:;) **4.** Practice

| **Practice** | **N (%)** | | |
| --- | --- | --- | --- |
|  | **Yes** | **No** | **Unclear/No impression** |
| Q1. Are you cooperating with the medical staff and following the guidance and education on ERAS? | 524(80.37) | 78(11.96) | 50(7.67) |
| Q2. Are you receiving nutritional support as advised by the medical staff? | 536(82.21) | 70(10.74) | 46(7.06) |
| Q3. Are you undergoing pre- and post-operative respiratory training as recommended by the medical staff? | 539(82.67) | 72(11.04) | 41(6.29) |
| Q4. Are you fasting from water and food for the duration prescribed by the medical staff? | 537(82.36) | 72(11.04) | 43(6.60) |
| Q5. Are you educated about pain scores and the use of analgesic pumps and medication by the medical staff? | 506(77.61) | 99(15.18) | 47(7.21) |
| Q6. Are you resuming water intake at the time recommended time by the medical staff during the post-operative period? | 556(85.28) | 69(10.58) | 27(4.14) |
| Q7. Are you resuming activities at the recommended time by the medical staff during the post-operative period? | 517(79.29) | 85(13.04) | 50(7.67) |
| Q8. Are you receiving thromboprophylaxis as recommended by the medical staff? | 540(82.82) | 73(11.20) | 39(5.98) |
